# Supplementary material for: Molecular Characterization of a Restriction Endonuclease PsaI from Pseudomonas anguilliseptica KM9 and Sequence Analysis of the PsaI R-M System
Source: Int J Mol Sci. 2025 Jul 8;26(14):6548. doi: 10.3390/ijms26146548 (PMC12294265; doi:10.3390/ijms26146548)
Supplement: Supplementary file 1 [file ijms-26-06548-s001.zip › ijms-3685572-supplementary.pdf]

Figure S1

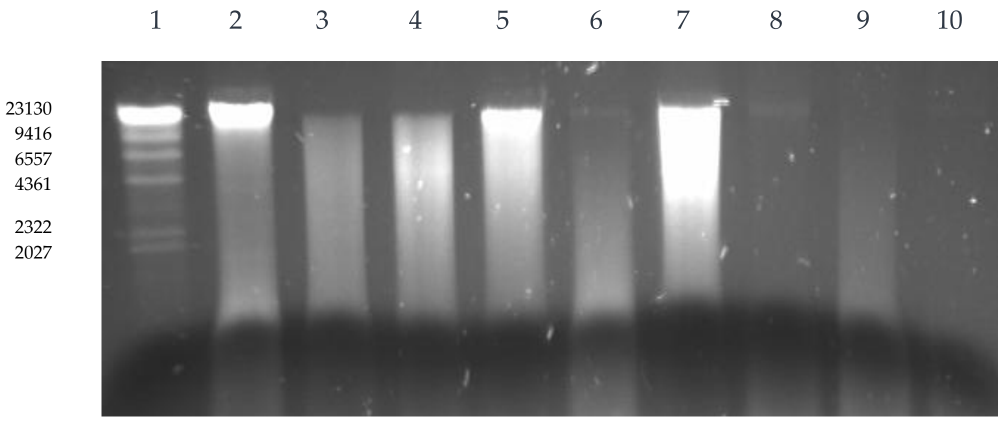

Figure S1. Bacteriophage lambda DNA digestion with endonucleases extracted from ten exemplary strains of bacteria. Lane 1 - crude bacterial extract of *P. anguilliseptica* KM9

Figure S2

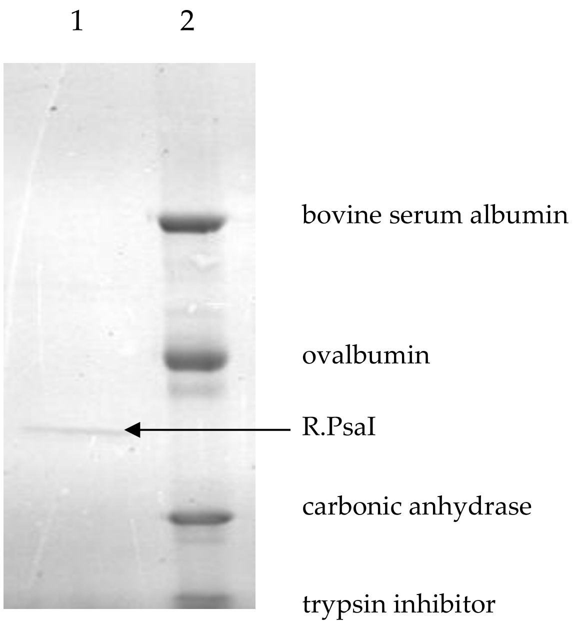

Figure S2. SDS-PAGE demonstrating the purity of restriction endonuclease PsaI. The gel was stained with Coomassie blue R-250. Lane 1, R.PsaI (200 ng of protein); lane 2, molecular mass markers: (a) bovine serum albumin *Mr* = 67 000; (b) ovalbumin *Mr* = 43 000; (c) carbonic anhydrase *Mr* =30 000; (d) trypsin inhibitor *Mr* =20 100, an arrow indicates the position of R.PsaI

Figure S3

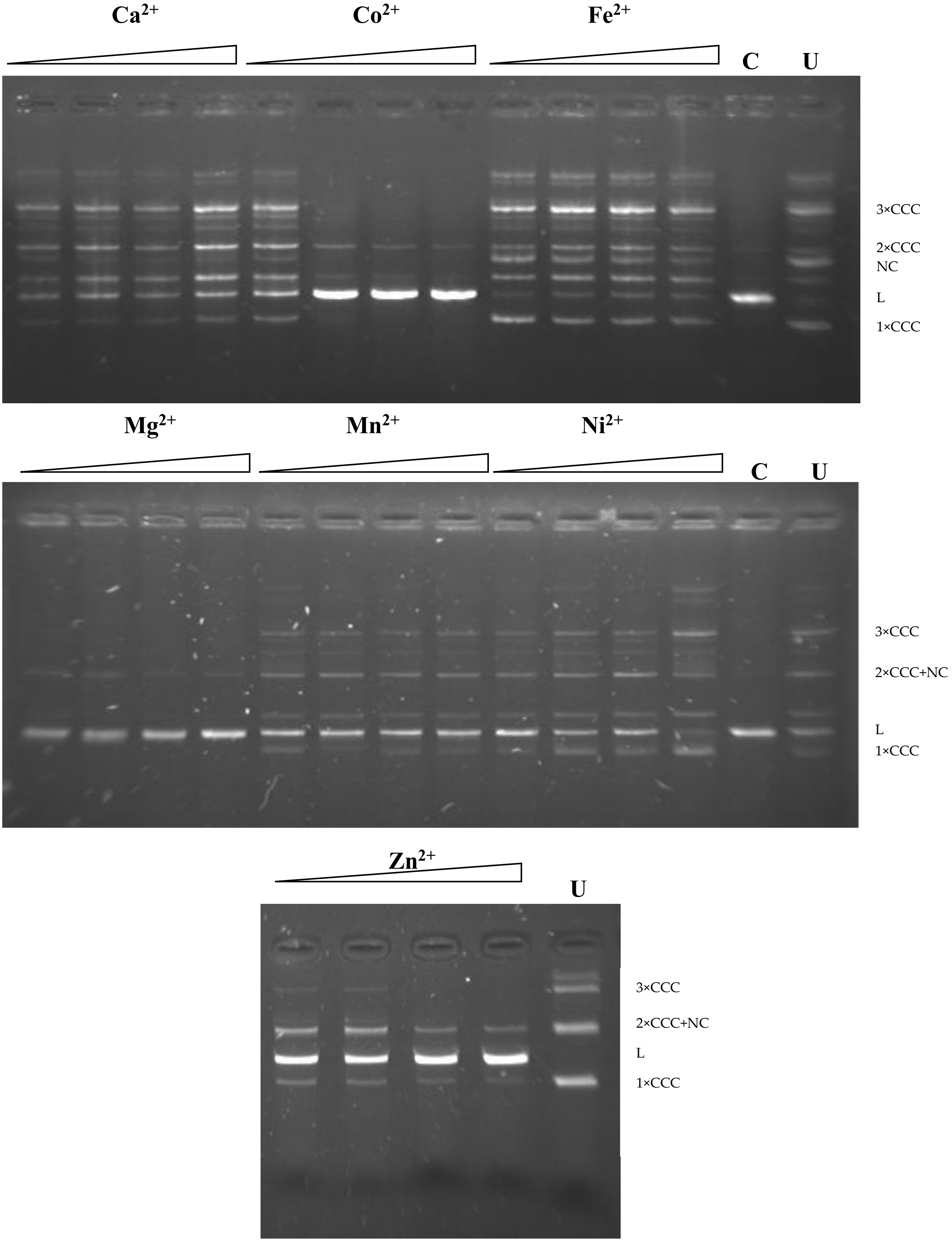

Figure S3. Cleavage of pUC18 DNA in the presence of an increasing amount (2.5, 5.0, 7.5, 10mM) of divalent metal ions. (Ca^{2+}, Co^{2+}, Fe^{2+}, Mg^{2+}, Mn^{2+}, Ni^{2+}, Zn^{2+}). C - control lane containing pUC18 digested with R.PsaI in NEB2 buffer (2686 bp), U - undigested pUC18 DNA, 3×CCC – supercoiled plasmid trimer, 2×CCC supercoiled plasmid dimer, 1×CCC supercoiled plasmid monomer, NC – nicked circular plasmid DNA, L – linear form of pUC18

Table S1. List of primers used for PCR and DNA sequencing.

Primer pair	Sequence (5'-3')	Source
GS5	ACATGTACACTGCGCGTAACCACCACAC	This study
GS6	AGATGTACAGTTTCCCGACTGGAAAG	
isometDIPY	TGATATYCCWTATGGBAT	This study
isometR	ACCACTWCCWGMAAAWGGRTC	
isoresinv1	ATAAAATTTGGCTTGATTT	This study
isoresinv2	GAGCAAGCAAGCAAGCTCACGC	
resPsastart	ATGCATGTGATCACTGAG	This study
metPsaend	CTATAGGTCGAACAATG	
isometinv1	TTGTCGTGGAGAACGTCCC	This study
isometF	GGAAAACCAATTAATGG	
recombinasePan	ATGTCCAGAACATTTGCG	This study
chelatasePan	GGTGGCCGCAGCCGGATC	
